# Sexual dimorphism during integrative endocrine and immune responses to ionizing radiation in mice

**DOI:** 10.1038/s41598-023-33629-7

**Published:** 2024-02-26

**Authors:** Marissa Burke, Kelly Wong, Yuli Talyansky, Siddhita D. Mhatre, Carol Mitchell, Cassandra M. Juran, Makaila Olson, Janani Iyer, Stephanie Puukila, Candice G. T. Tahimic, Lane K. Christenson, Moniece Lowe, Linda Rubinstein, Yasaman Shirazi-Fard, Marianne B. Sowa, Joshua S. Alwood, April E. Ronca, Amber M. Paul

**Affiliations:** 1 Department of Human Factors and Behavioral Neurobiology, Embry-Riddle Aeronautical University, Daytona Beach, FL 32114 USA; 2 Department of Physiology, Biophysics, and Systems Biology, Weill Cornell Medicine, New York, NY 10065 USA; 3 University of Texas Southwestern Medical Center, Dallas, TX 75390 USA; 4 Keck School of Medicine, University of Southern California, Los Angeles, CA 90033 USA; 5 KBR, Houston, TX 77002 USA; 6 Space Biosciences Division, NASA Ames Research Center, Moffett Field, CA 94035 USA; 7 Blue Marble Space Institute of Science, Seattle, WA 98104 USA; 8 Oak Ridge Associated Universities, Oak Ridge, TN 37830 USA; 9 Department of Biology, University of North Florida, Jacksonville, FL 32224 USA; 10 Department of Cell Biology and Physiology, University of Kansas Medical Center, Kansas City, KS 66160 USA; 11 Medical College of Wisconsin, Milwaukee, WI 53226 USA; 12 Universities Space Research Association, Mountain View, CA 94043 USA; 13 The Joseph Sagol Neuroscience Center, Sheba Research Hospital, Ramat Gan 52621, Israel; 14 Departments of Obstetrics & Gynecology, Wake Forest Medical School, Winston-Salem, NC USA

**Keywords:** Molecular medicine, Computational biology and bioinformatics, Computational models, Computational platforms and environments, Data acquisition, Data integration, Data publication and archiving, Databases, Gene ontology, Gene regulatory networks, Immunology, Adaptive immunity, Gene regulation in immune cells, Inflammation, Innate immune cells, Biomarkers, Diagnostic markers, Predictive markers, Prognostic markers, Endocrinology, Endocrine system and metabolic diseases, Medical research, Biomarkers, Experimental models of disease

## Abstract

Exposure to cosmic ionizing radiation is an innate risk of the spaceflight environment that can cause DNA damage and altered cellular function. In astronauts, longitudinal monitoring of physiological systems and interactions between these systems are important to consider for mitigation strategies. In addition, assessments of sex-specific biological responses in the unique environment of spaceflight are vital to support future exploration missions that include both females and males. Here we assessed sex-specific, multi-system immune and endocrine responses to simulated cosmic radiation. For this, 24-week-old, male and female *C57Bl/6J* mice were exposed to simplified five-ion, space-relevant galactic cosmic ray (GCRsim) radiation at 15 and 50 cGy, to simulate predicted radiation exposures that would be experienced during lunar and Martian missions, respectively. Blood and adrenal tissues were collected at 3- and 14-days post-irradiation for analysis of immune and endocrine biosignatures and pathways. Sexually dimorphic adrenal gland weights and morphology, differential total RNA expression with corresponding gene ontology, and unique immune phenotypes were altered by GCRsim. In brief, this study offers new insights into sexually dimorphic immune and endocrine kinetics following simulated cosmic radiation exposure and highlights the necessity for personalized translational approaches for astronauts during exploration missions.

## Introduction

Upcoming spaceflight missions will consist of long duration travel into the deep space environment, which will be taxing on the human body. Modeling cosmic radiation on Earth is challenging because it is difficult to simulate energy, particle distribution, and dose rates that are comparable to the deep space environment. Consequently, there is limited knowledge of unique deep space cosmic irradiation impacts on mammalian physiology. Yet, galactic cosmic ray (GCR) ionizing irradiation will be a major risk factor for astronauts on deep space missions and during lunar space tourism endeavors. Recently, NASA defined a dosing scheme for simulating deep space GCR (GCRsim), which is made up of space-relevant, high energy/charged particles consisting of multiple species of energetic particles that can be experienced during a single exposure^1^.

Immune dysfunction and adrenal hormone dysregulation are both consequences of low-Earth orbit environments on the International Space Station (ISS) and Shuttle missions^2,3^. The endocrine response, as it pertains to the acute stress hormone corticosterone (rodents) or cortisol (humans), has been studied extensively and clinically utilized as an anti-inflammatory countermeasure that promotes immune resolution^4^. However, in environments conducive to chronic stress, such as during long duration deep space missions, continuous production of stress hormones may become counterproductive, leading to possible immune dysregulation. Studies that determine the integrative communication networks between immune and endocrine physiological systems within the environment of deep space are limited to studies conducted during the Apollo era and short-term rodent flights outside the Van Allen belts^5,6^. Due to technical advances, GCRsim exposures have recently been developed by NASA Space Radiation Laboratory (NSRL). Therefore, the potential to examine the impact of this type of radiation on integrative physiological systems on ground are now possible.

Hormone synthesis and signaling are notably different between male and female humans^7^, including hormones of adrenal origin, such as cortisol, aldosterone, androgens, and epinephrine/norepinephrine^8^. Interestingly, disorders that involve the adrenal glands, including adrenocorticotropic hormone (ACTH)-independent Cushing’s syndrome (hypercortisolism) has a female bias^9^, with sex hormones playing a major role in influencing endocrine hormone activity^8^. In addition, autoimmune-related Addison’s disease caused by adrenal insufficiency also has higher prevalence in aged females (> 30 years)^8,10^, indicating age- and sex-specific regulation of immune function. In line with this, it is well-accepted that endocrine and immune responses are sexually-dimorphic, displaying complex kinetics^8,11,12^. Generally, females produce elevated inflammatory processes post-puberty, while males display elevated inflammation pre-puberty, indicating both sex hormone and age-related factors regulate immunity^13^. Furthermore, females produce elevated adaptive CD4^+^ T cells, whereas males produce elevated CD8^+^ T cells during adulthood, which may account for sexually distinct adaptive immune function^13^. Sex-dependent concentration of hormones, including androgens and estrogens, can regulate immunity through anti- or pro-inflammatory mechanisms^13,14^ and only recently has the relationship between the immune and endocrine physiological systems been considered^15^. Since, adrenal and immune responses are sexual dimorphic these variables should be evaluated during terrestrial and space medicine programs, as corticosteroids are routinely administered to treat inflammation^16^.

Upcoming exploration missions will expose crew to cosmic radiation. Thus, it is necessary to identify unique biosignatures produced in response to GCRsim radiation in order to better understand the physiological impacts of GCR. Generally, we hypothesize that immune and endocrine sexual dimorphism will be observed in response to radiation. Specifically, females may display pronounced immune activity in response to radiation that would be quickly resolved through elevated endocrine regulation. We assessed the effects of lunar and Martian GCRsim on both male and female mice to define the sex-specific immune and endocrine system responses to these unique doses. Collectively, male mice had smaller adrenal and immune organ sizes and reduced aldosterone levels that was coupled to distinct, sexually dimorphic immune responses. Longitudinal monitoring of immunity at 3- and 14-days post-irradiation identified sex-specific immune phenotypes, that are important for and apply to both space and terrestrial medicine programs that support mitigation strategies, personalized approaches, and biosignatures to treat long-term stress.

## Results

### Immune and endocrine systems display whole organ sexual dimorphism in structure, size, and function

**Figure 1:**
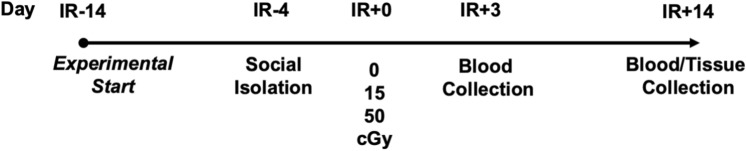
Representative experimental timeline. 24-week-old male and female *C57Bl/6J* were cage acclimated at 14-days prior to irradiation (IR-14) and socially isolated at IR-4. At IR + 0, mice were irradiated with 5-ion GCRsim ionizing radiation at 15 and 50 cGy, along with a unirradiated sham controls that were exposed to similar housing restraints and time as IR mice (0 cGy). Retro-orbital blood collection was performed on IR + 3 and abdominal aorta blood and tissues were collected on the time of euthanasia, IR + 14.

**Figure 2:**
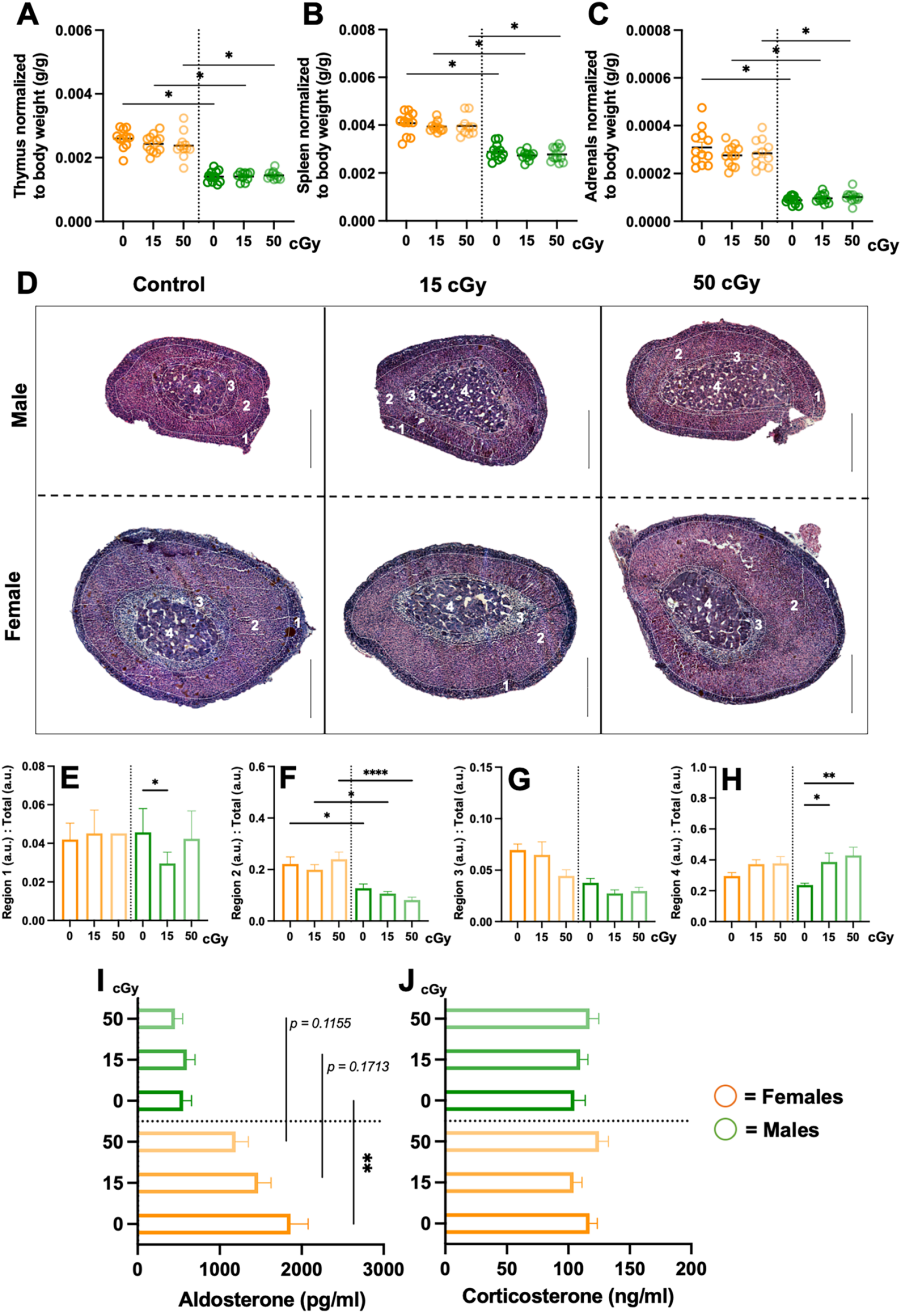
Sex-specific immune and endocrine system analysis, including adrenal gland architecture and function. Male and female mice were exposed to GCRsim (0, 15, and 50 cGy) and at 14-days post-irradiation mice were euthanized and immune thymus (**A**) and spleens (**B**) organs; and endocrine, adrenal glands (**C**) were isolated and weighed. All tissue weights were normalized to total body weights. 14-days post-irradiation glands were isolated, fixed, paraffin-embedded, sectioned (10 μm) and stained with hematoxylin and eosin for cellular architecture identification (**D**) and adrenal regional morphological size (**E**–**H**). Three-days post-irradiation blood was isolated and enzyme-linked immunosorbent assays (ELISA) were performed for adrenal hormone characterization, including aldosterone (**I**) and corticosterone (**J**). Scale bars = 450 μm. Sectioned regions highlight: 1. Zona glomerulosa; 2. Zona fasciculata; 3. Vacuolated zona fasciculata; and 4. Medulla. Females (orange) and males (green) are displayed. Parametric statistical analysis was performed on (**A**) and (**F**). Nonparametric analyses were performed on (**B**, **C**, **E**, **G**, **H**, **I**, and **J**), as described in the methods section. Weight data represent ± SEM, p* < 0.05, n = 10–12 per group. H&E data represent ± SEM, p* < 0.05, p** < 0.01, p**** < 0.001, n = 2–3 per group. Adrenal region images were scaled up and quantified with metric measurements denoted as arbitrary units (a.u.). ELISA data represent ± SEM, p** < 0.01, n = 10 per group with technical replicates (n = 3) performed with each ELISA.

To determine the effect of predicted lunar (15 cGy) and Martian (50 cGy) exploration mission radiation doses on immune and endocrine systems, male and female mice were exposed to acute, simplified 5-ion GCR simulated (GCRsim) radiation and multiple biosignatures were characterized at 3- and 14-days post-irradiation (Fig. 1). Tissue weights were recorded from select immune and endocrine tissues, including the thymus, spleen, and adrenal glands, which displayed sex-, but not GCRsim-dependent, reduction in males compared to females (Fig. 2A–C). In addition, hematoxylin and eosin staining revealed similar morphological disparities in male compared to female mice (Fig. 2D), including reduced size of the zona fasciculata region and marginally increased medulla sizes in males following radiation exposure (Fig. 2E–H). To determine if reduced adrenal size influences function, stress hormone levels were characterized in plasma collected 3-days post-irradiation. Regionally localized hormones produced from the zona glomerulosa (aldosterone, Fig. 2I) and the zona fasciculata (corticosterone, Fig. 2J) were analyzed, which revealed sexually dimorphic reduction of aldosterone in males verses females that was independent of radiation. Therefore, understanding sex-specific communication pathways between immune and endocrine systems are important in deciphering integrative responses that may be targets for future countermeasure development.

### Sexually dimorphic phenotypes of innate immunity

**Figure 3:**
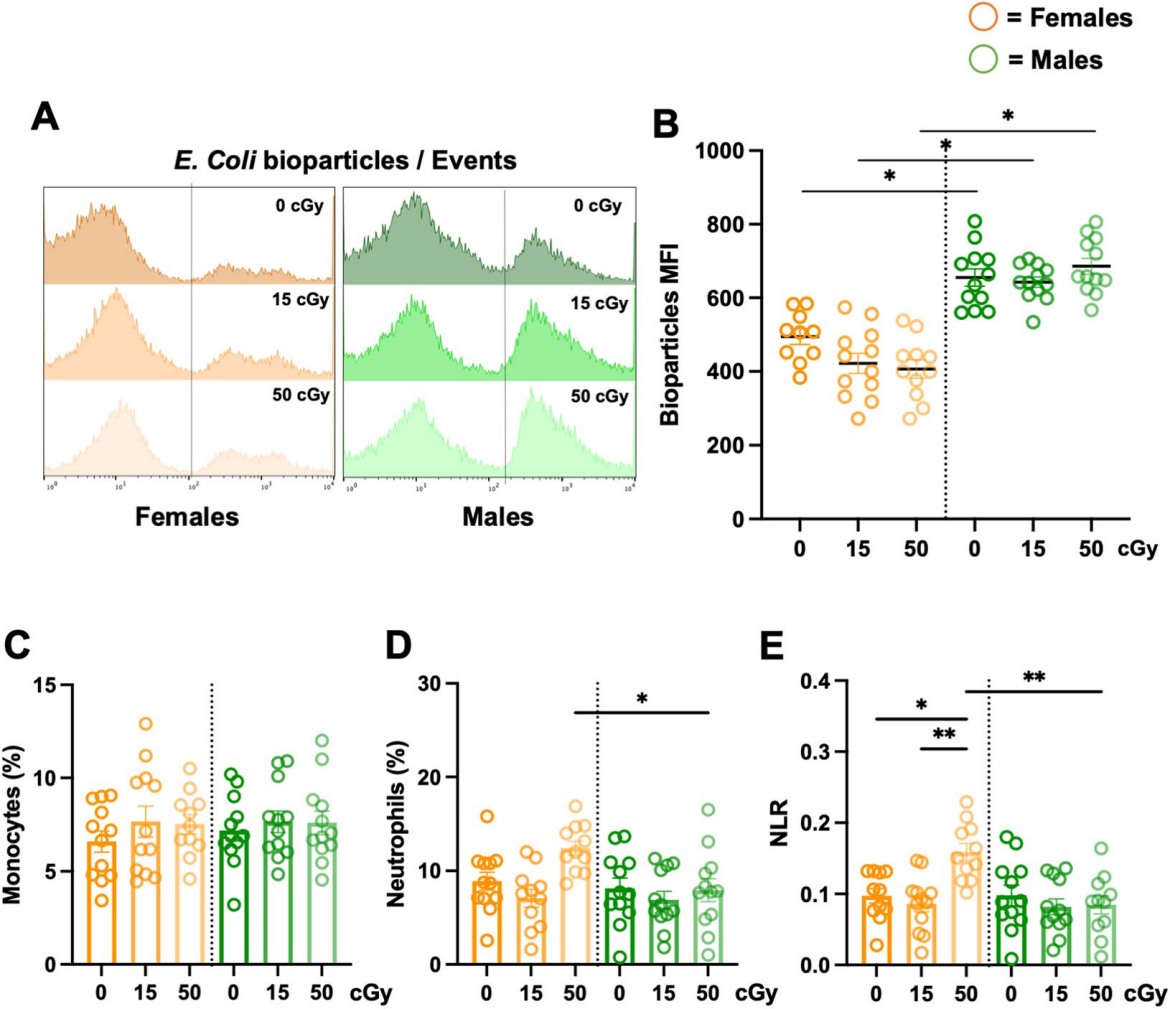
Sex-specific differences in phagocyte function, independent of phagocyte count. 24-week-old male and female *C57Bl/6J* mice were exposed to GCRsim (0, 15, and 50 cGy) and 3-days post-irradiation blood was collected. Red blood cells were lysed and leukocytes were stained and analyzed by flow cytometry. Phagocytic function was assessed using pH-sensitive *E.coli* bioparticles to determine the median fluorescence intensity (MFI) of phagocytosis (**A**, **B**). Cell count percentages (%) within total leukocytes (CD45^+^) were determined for monocytes (**C**), neutrophils (**D**), and neutrophil-to-lymphocyte ratio (NLR) (**E**). Parametric statistical analysis was performed on all data. Data represent ± SEM, p* < 0.05, p** < 0.01, n = 10–12 per group.

Reactive chemical species are known to be elevated in cells following radiation exposure^17–20^. However, exposure to GCRsim did not significantly change median fluorescence intensity (MFI) ROS concentrations at 3-days post-irradiation (sFig. 1A,B). We assessed phagocytic function at 3-days post-irradiation and found that males displayed robust (p < 0.05) phagocytic responses, compared to females, yet GCRsim exhibited no significant effect on phagocytosis (Fig. 3A,B). To confirm sexually dimorphic phagocytosis function was not a result of elevated cell number, phagocytic cell populations, monocytes, (Fig. 3C) and neutrophils (Fig. 3D) were identified. No significant difference in monocyte percentages were observed across sex or GCRsim doses, while neutrophil percentages in females were slightly elevated (p < 0.05) at 50 cGy, compared to males. Female mice also exhibited GCRsim dose-dependent elevation in neutrophil-to-lymphocyte ratio (NLR) at 3-days post-irradiation (Fig. 3E), with these values returning to control levels 14-days post-irradiation (sFig. 2). No change was observed in NLR in male mice. Collectively, these results indicate notable sexually dimorphic immune profiles are marginally influenced by GCRsim radiation.

### Longitudinal immune phenotypes display sexually dimorphic adaptive immunity

**Figure 4:**
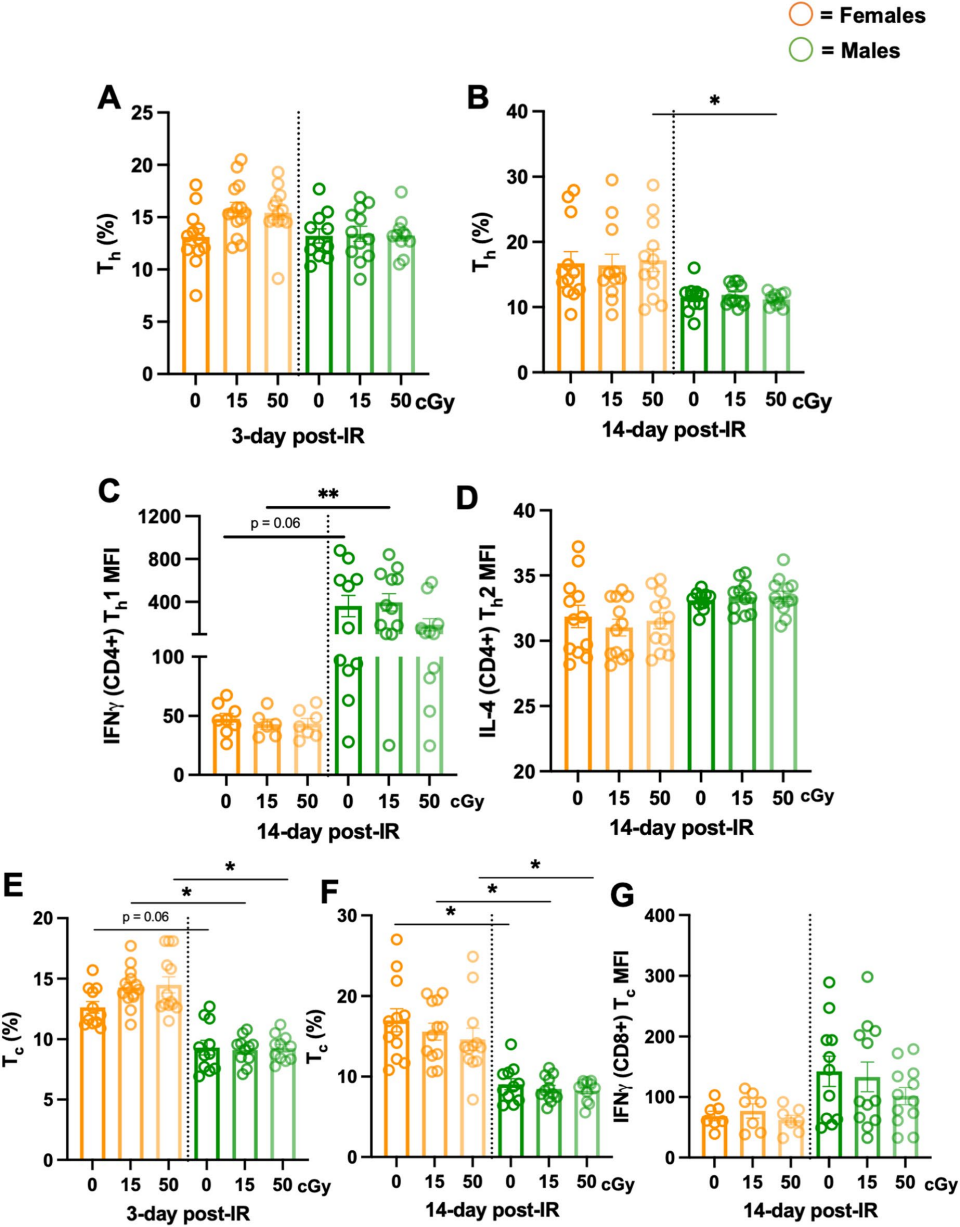
Longitudinal lymphocyte monitoring following irradiation displayed sex-specific effects. 24-week-old male and female *C57Bl/6J* mice were exposed to GCRsim (0, 15, and 50 cGy) and at 3- and 14-days post-irradiation, blood was collected. Red blood cells were lysed, leukocytes were stimulated for 16-h with a cell stimulation cocktail, stained, and analyzed by flow cytometry. Longitudinal monitoring of T_helper_ (T_h_, CD4^+^/CD3^+^) cell development percentages (%) post-irradiation in females (orange) and males (green) at day 3 (**A**) and day 14 (**B**) post-irradiation. T_h_ cell function assessed T_h_1 (IFNγ) median fluorescence intensity (MFI) (**C**) and T_h_2 (IL-4) MFI production (**D**) from CD4^+^/CD3^+^ lymphocytes. Longitudinal monitoring of T_cytotoxic_ (T_c_, CD8^+^/CD3^+^) cell development % post-irradiation in females and males at day 3 (**E**) and day 14 (**F**) post-irradiation. T_c_ cell function assessed (IFNγ) median fluorescence intensity (MFI) (**G**). Parametric statistical analysis was performed on (**A**) and (**D**). Nonparametric analyses were performed on (**B**, **C**, **E**, **F**, and **G**), as described in the methods section. Data represent ± SEM, p* < 0.05, p** < 0.01, n = 7–12 per group.

Since we previously observed sex-specific differences in mediators that are part of innate immunity, adaptive immunity was also profiled at 3- and 14-days post-irradiation. T helper cell percentages (T_h_, % CD4^+^/CD3^+^) did not display any significant differences at 3-days post-irradiation in females and males, however males displayed significantly reduced percentages compared to females at 14-days post-50 cGy irradiation (Fig. 4A,B). Interestingly, functional assessment via IFN_γ_ T_h_1 (CD4^+^) and IL-4 T_h_2 (CD4^+^) production at 14-days post-phorbol 12-myristate 13-acetate (PMA) and ionomycin stimulation, revealed males produced increased trends (p = 0.06, controls; p** < 0.01, 15 cGy) of IFN_γ_, without differences in IL-4 production (Fig. 4C,D), suggesting a T_h_1 bias in males compared to females. T cytotoxic cells (T_c_, % CD8^+^/CD3^+^) displayed significantly reduced population percentages in males at 3- and 14-days post-irradiation compared to females, across both irradiated groups (Fig. 4E,F). Due to notable sexual dimorphic disparities, T_cytotoxic_ function via IFN_γ_ production was measured, which revealed no significant differences across the sexes (Fig. 4G). Collectively, these results support longitudinal, sexually dimorphic adaptive immune phenotypes are observed independently of space-relevant radiation. Furthermore, these results highlight the necessity of defining sex-specific immune kinetics for personalized clinical programs in both space and on Earth.

### Blood and adrenal tissue transcriptomics reveal sex-specific responses following ionizing radiation

**Figure 5:**
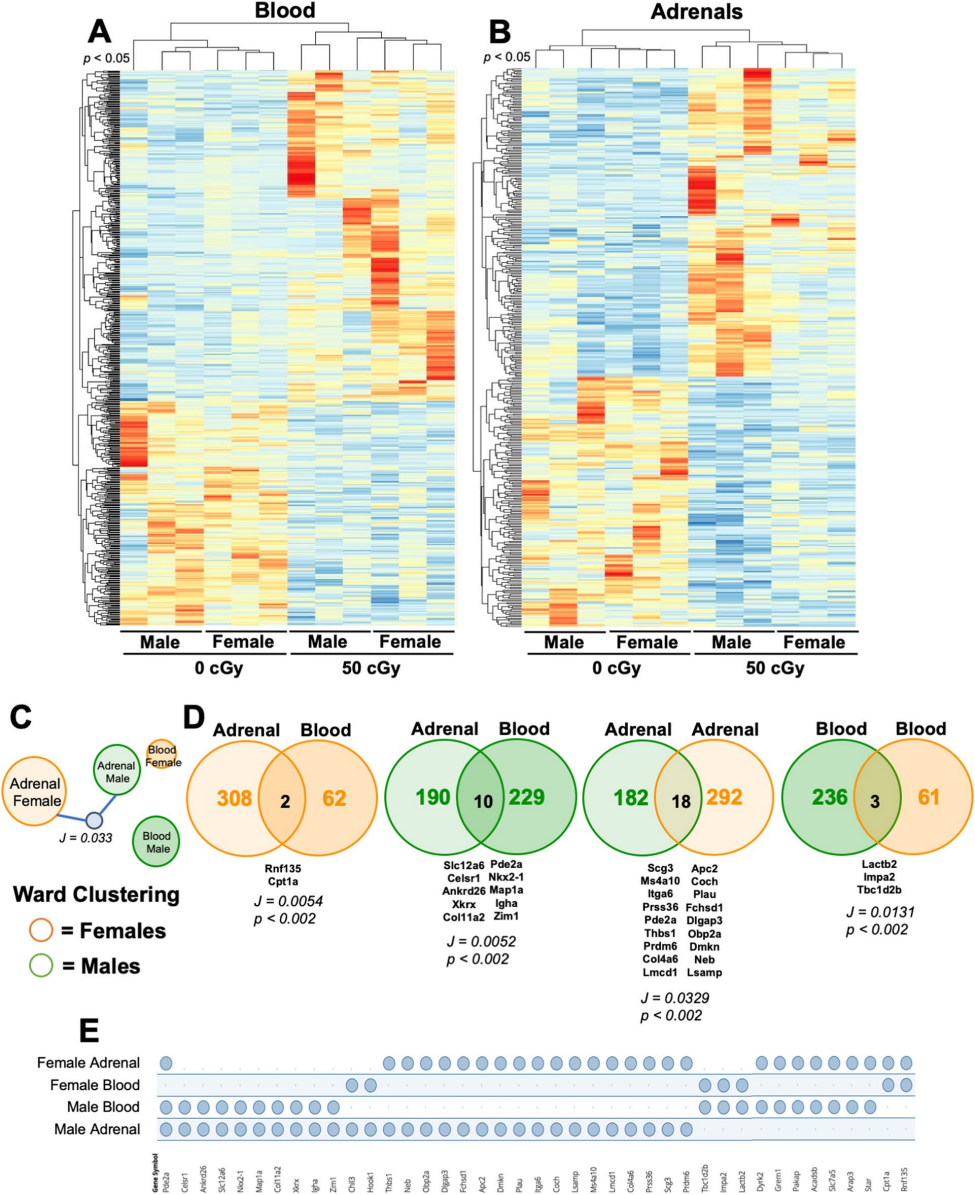
Sex- and dose-specific RNA sequencing profiles. Male and female mice were exposed to GCRsim (0 and 50 cGy) and at 14-days post-irradiation blood and adrenal glands were isolated and total RNA sequencing was performed. 50 cGy male and females were compared to 0 cGy sham controls. Heatmap of differentially expressed genes (DEG) in blood (**A**) and adrenal glands (**B**) from male and female mice exposed to 50 cGy versus 0 cGy controls (log_2_ fold change > 2 and p-value < 0.05). (**C**) Ward Clustering forceTree with Jaccard similarity child node (J = 0.033, p < 0.002) is displayed. (**D**) Venn diagrams of compared annotated datasets with overlapping Jaccard similarity score. (**E**) Jaccard similarity overlapping annotated genesets intersecting table displays overlapping genes across all tissue types and sexes. Clustering and geneset overlap (**E**) results generated using GeneWeaver and Venn diagrams (**D**) generated using Venny2.1**.** Data represent p < 0.05 (**A**, **B**) and p < 0.01 (**C–E**), n = 3 per group.

Since phenotypic profiles of immune and endocrine systems were largely limited to sex- not GCRsim differences, bulk RNA sequencing of male and female blood and adrenal tissues was performed on 14-days post-irradiation (0 and 50 cGy) samples. Heatmaps display unique, sex-specific differentially expressed genes (DEG, p < 0.05) biosignatures that cluster within control or radiation conditions in blood (Fig. 5A) and adrenals (Fig. 5B). Comparative analysis of DEG profiles within adrenals and blood indicated male and female adrenals clustered more readily than blood (p < 0.01, Fig. 5C), and Venn diagrams display some DEG overlap across each blood and adrenal tissue cohort (p < 0.01, Fig. 5D. Unique and overlapping DEG are shown (p < 0.01, Fig. 5E), indicating sexual dimorphism is evident within molecular biosignatures following simulated radiation in immune and endocrine systems. Therefore, providing evidence of discrete molecular pathways that may be targets for countermeasure developments.

**Figure 6:**
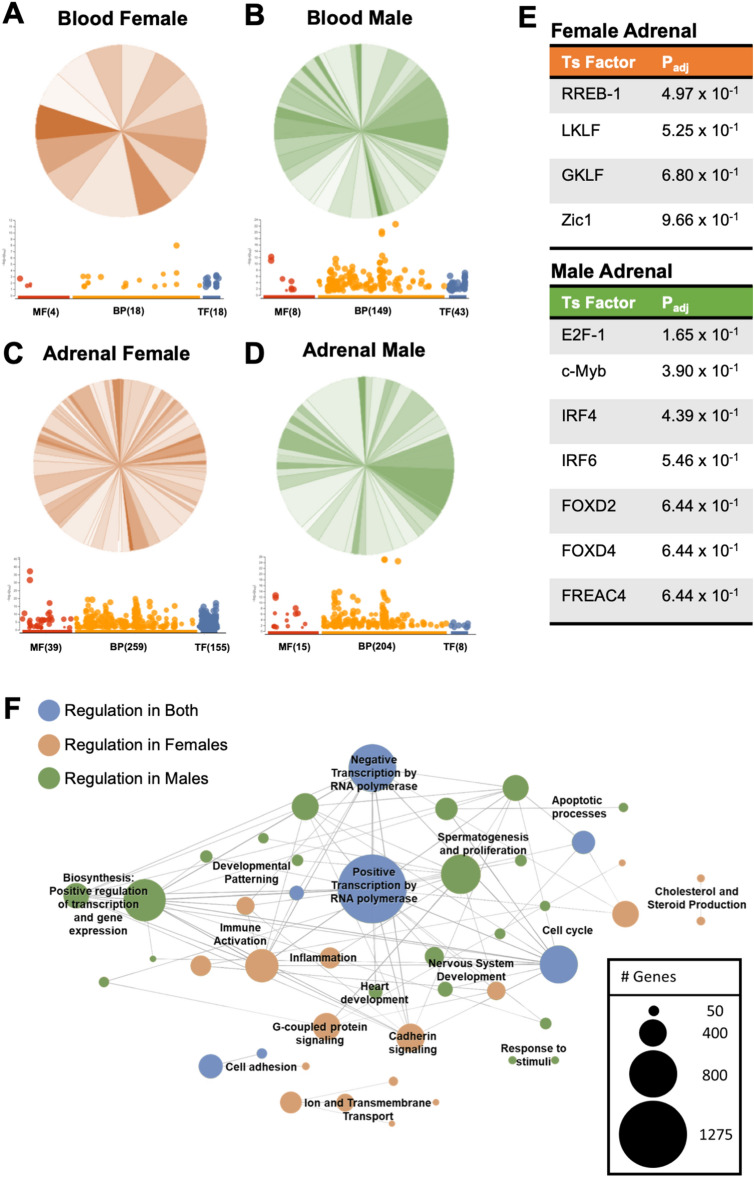
Adrenal and blood enriched gene ontology pathways are distinct and dimorphic. Male and female mice were exposed to GCRsim (0 and 50 cGy) and at 14-days post-irradiation blood and adrenal glands were isolated and total RNA sequencing was performed. 50 cGy male and females were compared to 0 cGy sham controls and enriched gene ontology PANTHER analysis converted gene to pathway (Venn Diagrams; shaded orange (female) and green (male) fractions all represent different PANTHER defined biological pathways and the number of genes involved in each pathway) and g:Profiler (dotplot; red denotes molecular function (MF), orange denotes biological pathways (BP), and blue denotes transcription factors (TF), and the number of genes identified in each) for annotated DEG (*p* < 0.01) in female blood (**A**) and adrenals (**C**), and male blood (**B**) and adrenals (**D**). Transcription factors analysis of female (orange label) and male (green label) adrenals, *p* < 0.01 (**E**). Overlapping blood and adrenal Consensus Pathways Analysis (CPA) for females (orange nodes) and males (green nodes) or both (blue nodes) and representative number of genes within each node. Significant pathways were identified as sharing at minimum one edge, three common genes, and a combined p-value < 0.01 (false discovery rate was not used for selection) (**F**). Data represent p < 0.01, n = 3 per group. PANTHER analysis orange circles denote biological pathways in female and green circles denote biological pathways in males.

Additionally, dimorphic responses of immune and endocrine systems were also described through enriched gene ontology pathways. Interpretive analysis indicates males display enhanced immune activation pathways in blood at day 14 post-irradiation compared to females (Fig. 6A,B and sFig. 3). Although male and female adrenals were diverse, sexually dimorphic pathways of interest were noted, including corticotropin-releasing hormone (CRH) receptor signaling and cholesterol biosynthesis pathways were enriched in females (Fig. 6C,D and sFig. 3), suggesting pronounced endocrine regulation pathways are engaged in females. g:Profiler pathway analysis indicated differences in select transcription factors in male and female adrenals (Fig. 6E). These results indicated male adrenals had additional immune activation signal transduction pathways triggered compared to females, including transcription factors interferon response factor (IRF)-4 and -6 (Fig. 6E). Finally, corrugated consensus pathway analysis (CPA) described sexual dimorphism in response to radiation and identified inflammation in females is efficiently regulated at day 14, in comparison to males, which produced random, disjointed inflammatory pathways that were non-resolved (Fig. 6F). Collectively, RNA sequencing analysis on immune and endocrine systems revealed molecular sex-specific differences in response to simulated radiation (50 cGy). Features include sexually dimorphic immune kinetics and organized endocrine hormone regulation of inflammation in females that was convoluted and non-resolved in males at 14-days post-irradiation.

## Discussion

With the advent of space tourism and inclusion of female crew for space exploratory class missions, it is critical to understand sexually dimorphic responses that are influenced by the space environment. Additionally, characterizing integrative physiological system crosstalk is also important for personalized directives applicable to both spaceflight and terrestrial medicine. In this study we assessed sexually dimorphic immune and endocrine responses to cosmic ionizing radiation. At discrete time points, simulated space-relevant GCR irradiation only marginally altered phenotypic immune and endocrine cellular biosignatures, while significant DEG determined by total RNA sequencing were identified. Females responded at early time points (day 3) post-irradiation via elevated NLR that was resolved by day 14 post-irradiation, yet males displayed no change in this immune biosignature. Conversely, males displayed robust DEG biosignatures and complex, yet disjointed, gene ontology pathways of immune activity until 14-days post-radiation, indicating delayed inflammatory resolution, which may be due to reduced endocrine production. Although cellular phenotypes showed marginal response to space-relevant ionizing GCR irradiation at both timeframes, discrete molecular analysis of DEG revealed sex-specific biological pathways following GCRsim irradiation. Additionally, notable baseline sex differences were observed in many biological biosignatures that were independent of radiation. For example, males displayed increased phagocytic function, T_h_1 IFN_γ_ production, and reduced T_cytotoxic_ percentages, adrenal size, morphology and hormone production. Collectively, these results suggest sexual dimorphism exists within endocrine and immune systems of mice, while exposure to space-relevant ionizing radiation altered some of these phenotypes. Therefore, careful monitoring and characterization of sexually dimorphic biosignatures that are longitudinal and kinetically altered should be considered for spaceflight mitigation strategies.

Immune and endocrine systems have several means of communication and cross-regulation as they are both connected via blood and lymphatic circulation^21^. The endocrine adrenal glands complete the hypothalamus–pituitary–adrenal (HPA) axis, otherwise known as the stress response. In mice, the adrenal medulla synthesizes and secretes the catecholamines, epinephrine and norepinephrine, as part of the sympathetic nervous system^22^. The mammalian adrenal cortex, typically consists of three distinct layers including, the zona glomerulosa, zona fasciculata, and zona reticularis (which is not clearly defined in mice)^23^. The adrenals produce aldosterone (water balance) and corticosterone (stress and metabolism)^24^. Due to a lack of the enzyme 17 alpha-hydroxylase (Cyp17A1), mice and rats do not produce androgens in their adrenals^25^. To determine irradiation dose depth effects of GCRsim on adrenal tissues, H&E was performed. Imaging revealed males had reduced adrenal overall and regional size disparities, yet no cellular irregularities were observed (Fig. 2A–H). Chronic physiological stress can influence thymus and spleen cellularity and weights^26^. In our study, reduced thymus and spleen weights were observed in males compared to females in all experimental conditions (Fig. 2A,B). These results align with others that have shown sex-specific disproportionate adrenal sizes and weights in rodents, with males displaying reduced adrenal glands^8,27^. However, further studies that address long-term effects of GCRsim-induced physiological stress on these tissues is an important future direction. Additionally, the zona fasciculata region (corticosterone synthesis) in females is larger than males, yet no differences were observed in corticosterone concentrations at 3-days post-irradiation (Fig. 2J). This may indicate corticosterone production is less pronounced in females following radiation. However, it is important to note that the timeframe of hormone analysis was performed at 3-days post-irradiation, which may miss the detection threshold of corticosterone. In line with this, the zona glomerulosa (aldosterone synthesis) region was not significantly different in size, yet aldosterone levels were elevated at baseline. Indeed, corticosterone dampens inflammation, while aldosterone promotes inflammation^4,28^. Further, aldosterone activates neutrophils^29^, promotes reactive oxygen species (ROS) production, which can enhance  inflammation^30^ and may in part contribute to elevated neutrophil populations and NLR in females (Fig. 3D,E) 3-days post-irradiation. Similarly, we recently characterized an elevated NLR as a robust indicator of low-grade inflammation and measure for immune monitoring in space and simulated-spaceflight models^31^, therefore females may produce robust immediate responses to ionizing radiation that are regulated by day-14 (sFig. 2). Crosstalk between stress hormones and neutrophils may serve as a primary mechanism for inflammatory regulation in females early post-irradiation, however, more studies that assess kinetic relationships between these unique immune and endocrine pathways are necessary. Additionally, RNA sequencing of multiple cell types within adrenal tissues and blood can provide additional information about cell-specific responses and are planned for future studies.

Aldosterone is a major regulator of blood pressure via promoting increased sodium reuptake into the vascular lumen and increased fluid volume^30^. Similar to our study, baseline aldosterone concentrations are generally elevated in female rodents and humans, which may make them more susceptible to cardiovascular disease^32^. However, it has also been reported that human and rodent males commonly have a higher risk of hypertension than females^33^. In addition, another report indicated basal serum levels of aldosterone are higher in males than females (3–11-weeks of age)^34^. Therefore, interesting dichotomies are involved with aldosterone kinetics, including dynamic changes in concentrations, age-associated kinetic profiles, diurnal collection timepoints, and inducible patterns of production of aldosterone (among other hormones) in response to blood pressure changes in the space environment. Therefore, additional studies assessing aldosterone concentrations that are associated with important metadata information are being considered in future experimental design.

The immune system consists of a complex network of cells and proteins that provide protection from internal and external cues. The innate immune system, or immediate responder, is nonspecific to pathogens or danger signals, with a response time that lasts up to a few weeks^35,36^. Active inflammation, ROS production, and phagocytosis of pathogens and dead cell debris are readily observed during this phase of immunity. Furthermore, elevated ROS are involved in mitochondrial dysfunction that is described in spaceflight^37^ and are critical mediators of granulocyte innate immunity^38^. Indeed, elevated reactive species production can be a function of the respiratory burst response of phagocytes^38,39^, which also corresponds with phagocytic function^39^. Interestingly, males displayed robust baseline phagocytic function compared to females that was independent of radiation (Fig. 3A,B). Similarly, elevated baseline phagocytosis in males versus females has been described in other studies^12,40^. Moreover, dampened phagocytic phenotypes in females may impact antigen presentation pathways and tolerance mechanisms. Indeed, disruption in tolerance processes are observed in autoimmunity^41^, which is more prevalent in females^8,42^. In addition, ROS, and other reactive species, can stimulate immune activity and is a product of functional phagocytosis^12,36,39^. Radiation only marginally elevated ROS production in males versus females at 3-days post-irradiation (sFig. 1A,B). Interestingly, at 14-days post-irradiation, oxidative stress GO pathways were identified in males, but not female blood. In addition, enrichment pathway analysis indicated corticotropin-releasing hormone (CRH) receptor signaling and cholesterol biosynthesis pathways were identified in females, but not males (Fig. 6A,B, sFig. 3). These results collectively indicate inflammatory profiles are efficiently regulated by day 14 post-irradiation in females compared to males, further supporting immune dimorphism between sexes, emphasizing differential immune recovery profiles, and confirming the necessity of physiological crosstalk to regulate inflammation^28,30^.

The adaptive system, or immune memory, has a delayed response of near 4–7 days. The bulk of these cells consist of T cells (T_helper_ and T_cytotoxic_) and B cells (antibody-producing cells)^36^. Adaptive T cell populations were phenotypically different in males and females, primarily at the 14-day post-irradiation timepoint. Upon closer examination, independent of radiation, the production of T_h_ lymphocytes at 3-day (females average, 14% and males average, 14%) and 14-day (females average, 18% and males average, 11%) suggests males may have impaired cell proliferation status or elevated apoptotic programs (Fig. 4A,B). Indeed, apoptotic programs were observed in male blood using PANTHER analyses and consensus pathways analysis (CPA) (Fig. 6F and sFig. 3). Lymphocyte phenotypes and functions were determined by expression levels of IL-4 (T_h_2), IFN_γ_ in CD4^+^ (T_h_1), and IFN_γ_ in CD8^+^ (T_c_) post-16-h stimulation with PMA and ionomycin. T_h_2 lymphocytes displayed no sex-specific differences in production of IL-4 (Fig. 4D). Interestingly, males produced an elevated trend in IFN_γ_ production in T_h_1 cells, with a significantly elevated difference at 15 cGy, compared to females. T_h_1 cells are responsible for enhancing phagocytic cell function and promoting inflammation^43^, which may correspond to inflammation-driven observations in gene pathway analyses at this age (28-weeks-old) and time point (14-day post-irradiation) in males (Fig. 6F and sFig. 3). Similar to human females that have higher CD4^+^ counts than males^13^, females produced elevated trends in T_h_ cell percentages at day 14, with significance reached at 50 cGy post-irradiation (Fig. 4B), Furthermore, T_c_ cells have similar IFN_γ_ production across sexes, but significantly reduced population percentages in males at day 3- and 14-post-irradiation (Figs. 4E,F). Other mediators of cytotoxic T cell function including granzyme and perforin were not characterized in this study but are considered for future studies. Collectively, adaptive immune phenotypes displayed sexually dimorphic responses, with minimal radiation-relevant differences at these time points post-exposure.

Total RNA sequencing results revealed sexual dimorphism within both blood and adrenals post-irradiation. Males display more differentially expressed genes (DEG = 239, p < 0.01) than females (DEG = 64, p < 0.01) in blood (Fig. 5C,D). Many DEG in males correspond with immune activity, such as B and T cell activation, oxidative stress, chemokine and cytokine signaling, and inflammation pathways (Fig. 6B, sFig. 3, and sTable 1). Since this was detected at 14-days post-radiation, this suggests male immune responses to space-relevant radiation are either delayed, dysregulated, or non-resolving, as similar activity was not observed in female blood (Fig. 6A, sFig. 3, and sTable 1). Both males and females display a large pool of adrenal DEG (310 = females, 200 = males, p < 0.01) with some overlapping similarities (Fig. 5C,D), however, females have more DEG corresponding with hormonal biosynthesis. For example, female adrenals displayed enrichment of cholesterol synthesis and corticotropin-releasing hormone (CRH) receptor pathways, whereas males did not (Fig. 6C,D, sFig. 3, and sTable 1). Adrenal transcription factors provided further insight into immune and endocrine sexual dimorphism. For instance, males display activated immune pathways at 14-day post-irradiation (c-Myb, IRF4, and IRF6, Fig. 6E). In contrast, females exhibit expression of RREB-1, GKLF (KLF4), and Zic1. These transcription factors are involved with p53 downregulation^44^. In line with this, adrenal cell turnover is significantly more rapid in females than males^45^. Additionally, GKLF (KLF4) is one of four Yamanaka factors (Oct3/4, Sox2, Klf4, c-Myc), which is involved in cellular reprogramming^46^. Thus, female adrenals may exhibit enhanced stemness, which may relate to immune and endocrine responses in females being more organized, responsive, and adaptable to external and internal cues. Collectively, sex-specific, dynamic DEG are expressed 14-days post-irradiation in response to space-relevant doses of simulated ionizing radiation in both immune and endocrine systems.

In this study we assessed sexually dimorphic immune and endocrine responses to cosmic ionizing radiation. Based on foreseen immune and endocrine deficits, the central nervous system most likely plays a significant role in crosstalk regulation, which requires further studies. Additionally, the involvement of sex hormones on adrenal and immune function adds an additional layer of complexity during integrative crosstalk, which were not assessed in this manuscript. However, the impact of ovarian steroids and the estrous cycle of female mice^47^ is also a future consideration for experimental interpretation. Generally, females produced rapid selective immune responses and resolve compared to males in blood, along with contributing adrenal hormone influence. In short, this study identified notable immune and endocrine integrative pathways that are sexually dimorphic. Advances in this research can direct personalized medicine and pharmaceutical approaches for future exploration and commercial missions.

## Methods

### Ethics statement

All animal experimental procedures were approved by the Institutional Animal Care and Use Committee (IACUC) at Brookhaven National Laboratory (BNL) protocol number 516. All experiments were performed by trained personnel in AAALAC accredited animal facilities at BNL, while conforming to the U.S. National Institutes of Health Guide for the Care and Use of Laboratory Animals. This study is reported in accordance with ARRIVE guidelines.

### Animal studies

24-week-old *C57Bl/6J* male and female mice were purchased from Jackson Laboratory (CA, USA) and shipped to Brookhaven National Laboratory (BNL) 14-days prior to irradiation (IR-14). At arrival to BNL, mice were quarantined and acclimated to a 12 h light:12 h dark cycle at standard temperature (23 ± 2 °C) and humidity (approximately 50%). Food and water were given ad libitum, and bedding was changed weekly. At IR-4, mice were socially isolated, and, at IR + 0, mice irradiated with the 5-ion galactic cosmic ray radiation (GCRsim) at 0, 15, and 50 cGy doses. Mice were weighed at euthanasia. At IR + 3, blood was collected by retro-orbital sampling (isoflurane anesthesia, between 11:00–14:00 h) and at IR + 14 mice were euthanized by CO_2_ overdose. Blood was collected through the abdominal aorta (11:00–16:00 h) and adrenal, thymus, and spleen tissues were isolated and weighed. Blood samples were centrifuged and separated cell and plasma fractions were either stored at – 80 °C (blood cell aliquot and plasma) until future processing or immediately stimulated and analyzed by flow cytometry (blood cell aliquot). Adrenals, spleen, and thymus were either flash frozen in liquid nitrogen with subsequent storage at – 80 °C or were overnight fixed in 4% paraformaldehyde (PFA), followed by transfer and storage in 1 × phosphate buffered saline (PBS) and stored at 4 °C until paraffin-embedding and sectioning. The experimental timeline is described in Fig. 1.

### Simulated GCR irradiation

On day IR-1, mice were transferred to the NASA Space Radiation Laboratory (NSRL) on BNL’s campus by animal care staff. On day IR + 0, mice received whole-body irradiation using the simplified 5-ion GCRsim irradiation scheme (protons at 250 and 1000 MeV, ^28^Si at 600 MeV/n, ^4^He at 250 MeV/n, ^16^O at 350 MeV/n, and ^56^Fe at 600 MeV/n) with 15 and 50 cGy, while 0 cGy sham controls were also performed in similar housing condition, in the absence of irradiation. GCRsim ions, energies, and doses were determined by a NASA consensus formula as previously described^1,49,50^. Beam time exposures ranged from ~ 15 min (15 cGy) to 35–45 min (50 cGy) per n = 12 batched cohorts.

### Blood collections

Blood was collected in 0.5 M EDTA coated tubes and centrifuged at 2000×*g* for 15 min in a 4 °C refrigerated centrifuge. Plasma was separated and blood sample aliquots (50 μl) were stored at – 80 °C. Remaining blood cells were lysed with 1 × red blood cell (RBC) lysis buffer (Thermo Fisher Scientific) and resuspended in complete media (RPMI + 10% FBS and 1% P/S). Samples were stored on ice for further processing.

### Bioparticle phagocytosis assay

*Escherichia coli* bioparticles (0.2 mg/ml) were incubated in complete media at 37 °C for 30 min following manufacturer's recommendations. Prior to flow cytometry, SYTOX™ live/dead (0.5 μM) was added and cells were analyzed. CellROX™, SYTOX™, and *E. coli* bioparticles were purchased from Thermo Fisher Scientific.

### Stimulation assay

Collected and lysed whole blood containing leukocytes were cultured in 96-well U-bottom plates at 1 × 10^5^ cells/well. Cell stimulation mix cocktail (500 ×) containing phorbol 12-myristate 13-acetate (PMA) and ionomycin (Thermo Fisher Scientific) was added to the wells at 1 × final concentration and cells were incubated at 37 °C for 16 h. After stimulation, supernatant was collected and store at − 80 °C and cells were fixed and probed for flow cytometric analysis.

### Flow cytometry

Leukocytes were fixed with 2% paraformaldehyde (PFA), washed with 1 × PBS, Fc blocked with anti-CD16 (1:20), and probed with CellROX™, SYTOX™ live/dead stain, anti-CD45, anti-CD11b and anti-Ly6G, anti-CD3, anti-CD4, anti-CD8, anti-IFNγ, and anti-IL-4. Single-stain and unstained controls were also prepared and all cells incubated for 1-h at room temperature in the dark. After incubation, cells were washed and resuspended in 1 × PBS and 30,000 events/sample were acquired using a SONYH800 or Becton Dickinson FACS Calibur flow cytometers. FlowJo (version 10.5.3) was used for data analysis. All antibodies and dyes were purchased from Thermo Fisher Scientific.

### Paraffin-embedding protocol

Adrenal tissues were transferred to tissue embedding cassettes and processed through dehydration steps prior to paraffin embedding. Tissue blocks were sectioned on a Leica microtome (10 μm) and mounted on Superfrost Plus microscope slides (Fisher Scientific). Blinded IDs were labelled on each slide for subsequent analysis.

### Hematoxylin and eosin (H&E) assay

Slides were deparaffinized and rehydrated with xylene and ethanol (100–30%) and rinsed in deionized (DI) water. Slides were removed from water and hematoxylin was applied and incubated for 5 min. Slides were rinsed with DI water and bluing reagent was applied for 10–15 s, rinsed with DI water, then soaked in 100% ethanol for 10 s. Eosin Y solution was applied for 2–3 min and slides were dehydrated in 3 changes of 100% ethanol for 1–2 min. Slides were then mounted with VectaMount® mounting media and coverslips and set overnight to dry.

### Enzyme-linked immunosorbent assay

Plasma isolated from mice at 3-days post-irradiation were analyzed for aldosterone and corticosterone by an Enzyme-linked Immunosorbent Assay (Abnova). Protocol was performed following manufacturers’ recommendations.

### RNA sequencing

Total RNA was extracted with Trizol (Thermo Fisher) and purified using Quick-RNA Miniprep Kit (Zymo Research). RNA-sequencing libraries were performed using Illumina’s total RNA kit per manufacturer’s instructions. RNA integrity was determined using an Agilent TapeStation with eRIN values > 7.500 ng of total RNA was used as input. rRNA was depleted, cDNA (first and second strands) were synthesized, and adaptor index ligation and strand selection was performed (Illumina Stranded total RNA Prep with Ribo-Zero Plus). For multiplexing, barcodes with unique indices out of 96, were used per sample (IDT for Illumina). Libraries were amplified by PCR on a Mastercycler Pro (Eppendorf) and purified with RNAClean XP Agencourt beads (Beckman Coulter). Libraries were sequenced on a NextSeq1000 (Illumina) to generate 30 M, 75-bp paired end reads per sample.

### Differential expression and pathway enrichment analysis

Raw reads were assessed for quality and trimmed using FastQC^51^. Passing reads were then quasi-mapped to Ensembl release GRCm39 using Salmon v1.9.0^52,53^ and imported into R using tximports^54,55^. Normalized gene expression analysis of male and female adrenal and/or blood 50 cGy radiation exposure compared to controls were conducted using DESeq2 and significance presented for genes identified to have greater than log_2_ fold change of 2 and a cutoff p-value of less than 0.05 (p values were multiple test corrected using the Benjamini–Hochberg method). Visualization of DEG were prepared using the pheatmap function of the DESeq results matrix.

Pathways analysis was conducted using several toolkits that utilize DEG lists including Panther and g:Profiler, and Gene Ontology (GO) Consensus Pathway Analysis (CPA) toolkit^56^ which considered both gene and Log_2_ fold change between 50 cGy radiation exposure and control in males and females.

### Statistical analysis

Data outliers were identified using a Grubb’s test (alpha = 0.05) and removed. A normality and lognormality test to assess normal Gaussian distribution, with Kolmogorov–Smirnov normality test (p < 0.05) was performed. If data passed normality a parametric analysis was performed using a paired one-way ANOVA with a Tukey post hoc test. If data did not pass normality a nonparametric analysis was performed using a paired analysis and a Dunn’s multiple comparisons post hoc test. All statistical analyses were performed using GraphPad Prism software (version 6.0).

### Supplementary Information

Supplementary Information 1.

Supplementary Table 1.

## Data Availability

All RNA sequencing raw data and metadata are available in the NASA Open Science data repository (OSDR)^48^. OSDR identifier is OSD-566. 10.26030/s8vj-6k50.
