# Supplementary material for: Sexual dimorphism during integrative endocrine and immune responses to ionizing radiation in mice
Source: Sci Rep. 2024 Feb 26;14:7334. doi: 10.1038/s41598-023-33629-7 (PMC10897391; doi:10.1038/s41598-023-33629-7)
Supplement: Supplementary file 1 — Supplementary Information 1. [file 41598_2023_33629_MOESM1_ESM.docx]

**Supplemental Material for**

**Sexual Dimorphism during Integrative Endocrine and Immune Responses to Ionizing Radiation in Mice**

Burke et al., 2023

Correspondence to: [amber.paul@erau.edu](mailto:amber.paul@erau.edu)

**Supplementary Methods**

**Blood collections**

Blood was collected in 0.5 M EDTA coated tubes and centrifuged at 2000 x g for 15 minutes in a 4ºC refrigerated centrifuge. Plasma was separated and blood sample aliquots (50 μl) were stored at -80ºC. Remaining blood cells were lysed with 1x red blood cell (RBC) lysis buffer (Thermo Fisher Scientific) and resuspended in complete media (RPMI + 10% FBS and 1% P/S). Samples were stored on ice for further processing.

**Reactive Species assay**

Reactive species were analyzed by staining live cells with CellROX™ and SYTOX™ live/dead stains for 1-hour following manufacturers recommendations. Briefly, positive TBHP (50 μM) and unstained controls were prepared concurrently with all samples. CellROX™ (1 μM) was added to the positive control and all samples were incubated at 37ºC for 1 hour with caps open to allow airflow to cells. Prior to flow cytometry, SYTOX™ live/dead (0.5 μM) was added and cells were analyzed.

**Flow cytometry**

Leukocytes were fixed with 2% paraformaldehyde (PFA), washed with 1x PBS, and Fc blocked with anti-CD16 (1:20), and probed with CellROX™, SYTOX™ live/dead stain, anti-CD45, anti-CD11b and anti-Ly6G, anti-CD3, anti-CD4, anti-CD8, anti-IFN, and anti-IL-4. Single-stain and unstained controls were also prepared and all cells incubated for 1-hour at room temperature in the dark. After incubation, cells were washed and resuspended in 1x PBS and 30,000 events / sample were acquired using a SONYH800 or a Becton Dickinson FACS Calibur flow cytometers. FlowJo (version 10.5.3) was used for data analysis. All antibodies and dyes were purchased from Thermo Fisher Scientific.

**RNA sequencing**

Total RNA was extracted with Trizol (Thermo Fisher) and purified using Quick-RNA Miniprep Kit (Zymo Research). RNA-sequencing libraries were performed using Illumina’s total RNA kit per manufacturer’s instructions. RNA integrity was determined using an Agilent TapeStation with eRIN values > 7. 500 ng of total RNA was used as input. rRNA was depleted, cDNA (first and second strands) were synthesized, and adaptor index ligation and strand selection was performed (Illumina Stranded total RNA Prep with Ribo-Zero Plus). For multiplexing, barcodes with unique indices out of 96, were used per sample (IDT for Illumina). Libraries were amplified by PCR on a Mastercycler Pro (Eppendorf) and purified with RNAClean XP Agencourt beads (Beckman Coulter). Libraries were sequenced on a NextSeq1000 (Illumina) to generate 30 M, 75-bp paired end reads per sample.

**Differential expression and pathway enrichment analysis**

Raw reads were assessed for quality and trimmed using FastQC (*51*). Passing reads were then quasi-mapped to Ensembl release GRCm39 using Salmon v1.9.0 (*52, 53*) and imported to R using tximports (*54, 55*). Normalized gene expression analysis of male and female adrenal and/or blood 50cGy radiation exposure compared to control was conducted using DESeq2 and significance presented for genes identified to have greater than log_2_ fold change (L2FC) of 2 and discovery rate cutoff p-value of less than 0.05 (p values were multiple test corrected using the Benjamini–Hochberg method). Visualization of DEG were prepared using the pheatmap function of the DESeq results matrix.

Pathways analysis was conducted using several toolkits that utilize DEG lists including Panther and g:Profiler, and Gene Ontology (GO) Consensus Pathway Analysis (CPA) toolkit (*56*) which considered both gene and Log_2_ fold change between 50 cGy radiation exposure and control.

**Statistical Analysis**

Data outliers were identified using a Grubb’s test (alpha = 0.05) and removed. Normality and lognormality test to assess normal Gaussian distribution, with Kolmogorov-Smirnov normality test (p < 0.05) was performed. If data passed normality a parametric analysis was performed using a paired one-way ANOVA with a Tukey post hoc test. If data did not pass normality a nonparametric analysis was performed using a paired analysis and a Dunn’s multiple comparisons post hoc test. All statistical analyses were performed using GraphPad Prism software (version 6.0).

**Supplementary Figures**

**Supplemental Table 1.** Differentially expresses gene (DEG) annotated gene lists for male and female blood and adrenals (p < 0.05).


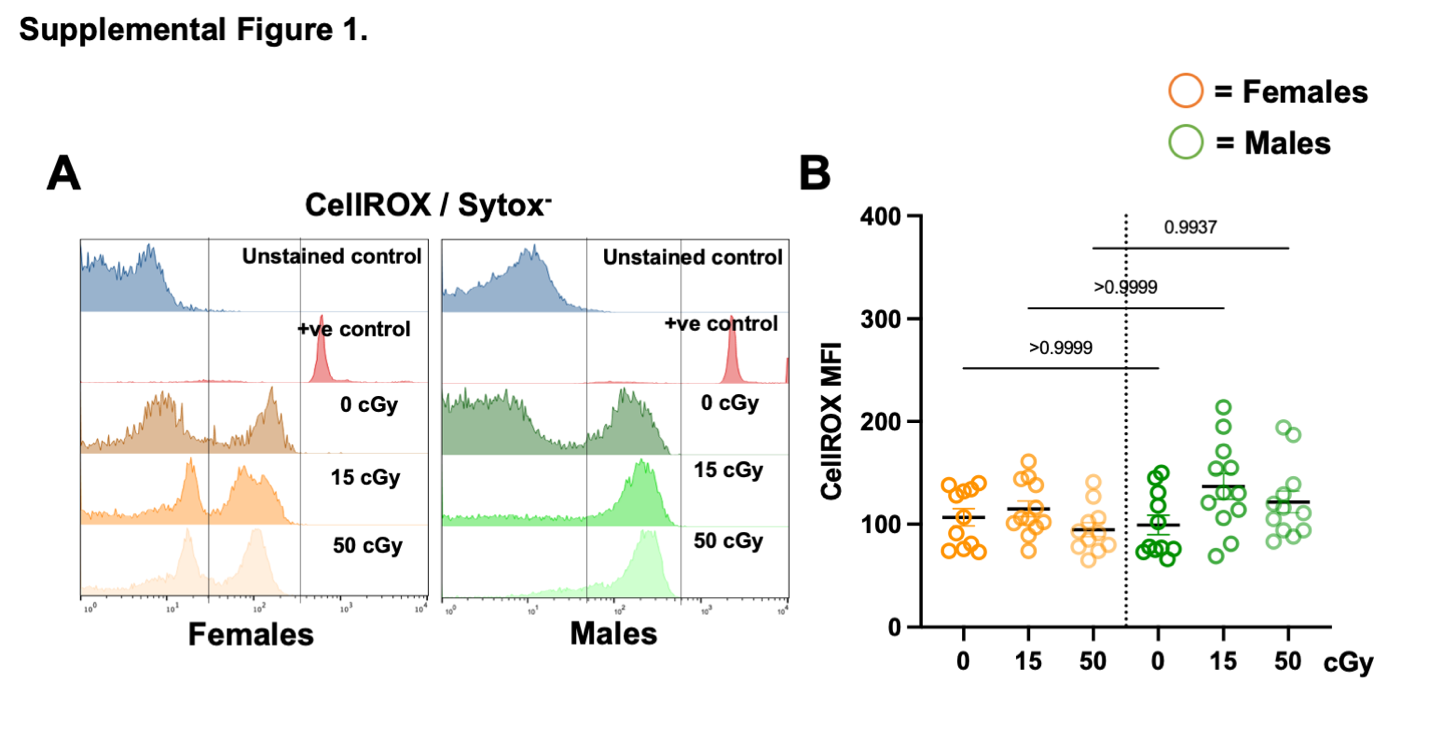
**Supplemental** **Figure 1. Sex-specific trends in oxidative stress.** 24-week-old male and female *C57Bl/6J* mice were exposed to GCR_sim_ (0, 15, and 50 cGy) and 3-days post-irradiation blood was collected. Red blood cells were lysed and leukocytes were stained and analyzed by flow cytometry. Representative histogram **(A)** of reactive species that measured the median fluorescence intensity (MFI) CellROX^+^ Sytox^-^ (live cells) in female (orange) and male (green) samples **(B)**. Nonparametric analyses were performed on Figure 3B, as described in the methods section. Data represent ±SEM, n = 10-12 per group.

**
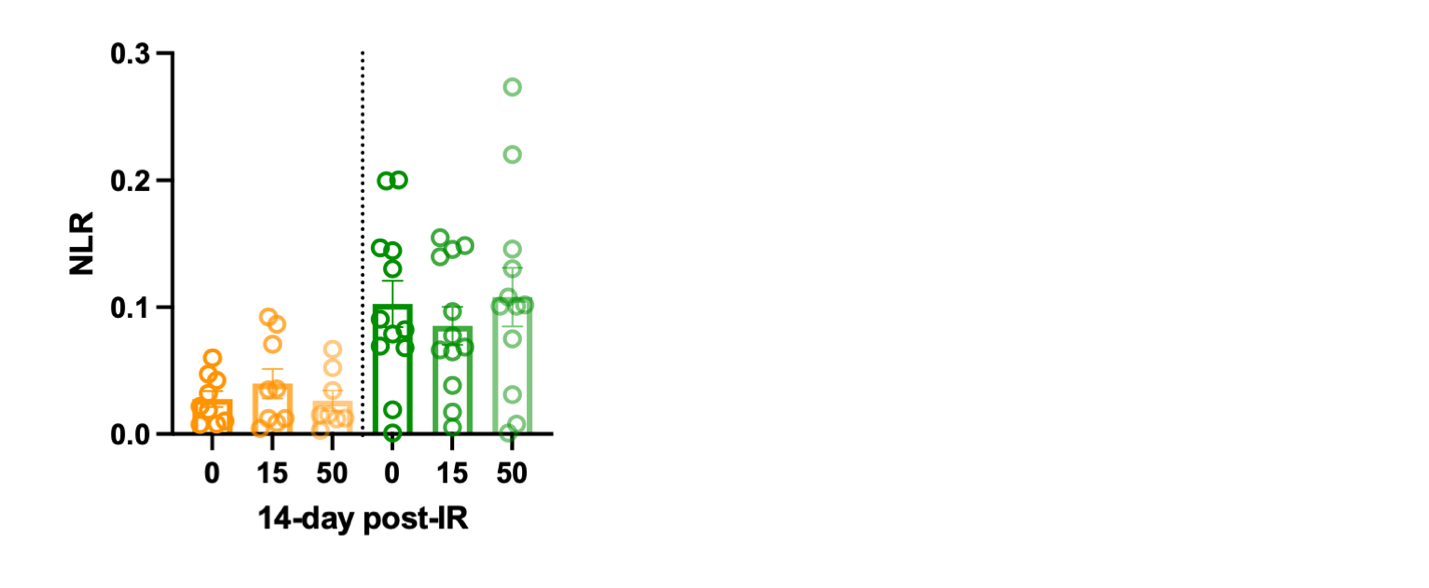
**

**Supplemental Figure 2. Minimal difference in NLR at 14-days post-irradiation.** 24-month-old male and female *C57Bl/6J* mice were exposed to GCR_sim_ (0, 15, and 50 cGy) and 14-days post-IR blood was collected. Cells were lysed and leukocytes were stained and analyzed by flow cytometry. Neutrophil-to-lymphocyte ratio (NLR) was identified. Nonparametric analysis was performed, as described in the methods section. Data represent ±SEM, p* < 0.05, n= 10-12 per group

**
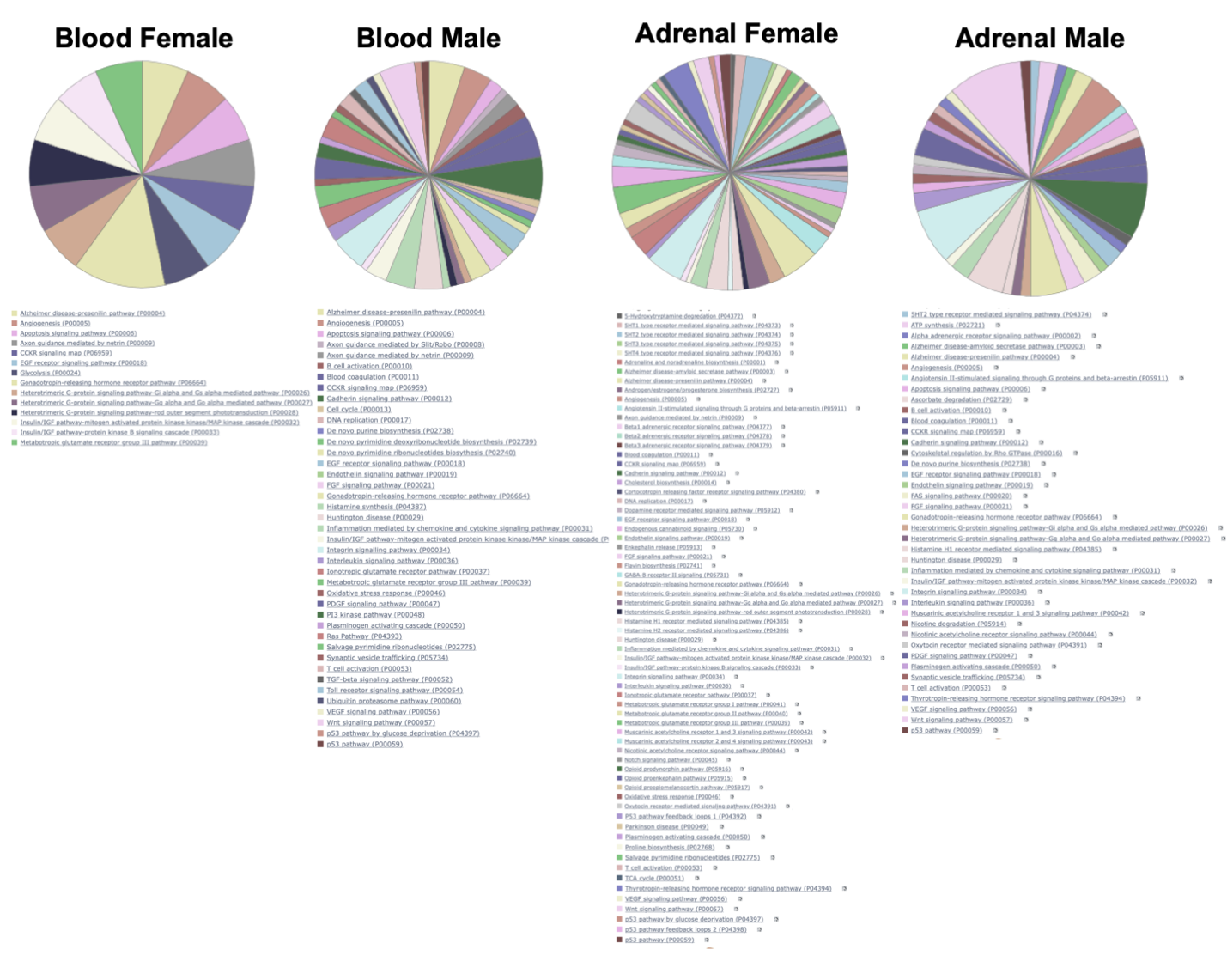
**

**Supplemental Figure 3. Adrenal and blood enriched gene ontology pathways are distinct and dimorphic.** Male and female mice were exposed to GCR_sim_ (0 and 50 cGy) and at 14-days post-irradiation blood and adrenal glands were isolated and total RNA sequencing was performed. 50 cGy male and females were compared to 0 cGy sham controls and Enriched Gene Ontology PANTHER analysis converted gene to pathway for annotated DEG (*p < 0.01*) in female blood **(A)** and adrenals **(C)**, and male blood **(B)** and adrenals **(D)**. Data represent p* < 0.01, n= 3 per group. PANTHER analysis shaded circles denote unique biological pathways listed below each circle in female and males.
